# Albumin-fused thioredoxin ameliorates high-fat diet-induced non-alcoholic steatohepatitis

**DOI:** 10.1016/j.heliyon.2024.e25485

**Published:** 2024-02-02

**Authors:** Ryota Murata, Hiroshi Watanabe, Ryotaro Iwakiri, Mayuko Chikamatsu, Takao Satoh, Isamu Noguchi, Kengo Yasuda, Ayano Nishinoiri, Takuma Yoshitake, Hiroto Nosaki, Hitoshi Maeda, Toru Maruyama

**Affiliations:** a Department of Biopharmaceutics, Graduate School of Pharmaceutical Sciences, Kumamoto University, 5-1 Oe-Honmachi, Chuo-ku, Kumamoto 862-0973, Japan; b Kumamoto Industrial Research Institute, Kumamoto, Japan

**Keywords:** Albumin fusion, Thioredoxin, Non-alcoholic fatty liver disease, Non-alcoholic steatohepatitis, Oxidative stress, Inflammation, Apoptosis

## Abstract

The pathogenesis of non-alcoholic steatohepatitis (NASH) involves the simultaneous interaction of multiple factors such as lipid accumulation, oxidative stress, and inflammatory response. Here, the effect of human serum albumin (HSA) fused to thioredoxin (Trx) on NASH was investigated. Trx is known to have anti-oxidative, anti-inflammatory, and anti-apoptotic effects. However, Trx is a low molecular weight protein and is rapidly eliminated from the blood. To overcome the low availability of Trx, HSA-Trx fusion protein was produced and evaluated the therapeutic effect on high-fat diet (HFD)-induced NASH model mice. HSA-Trx administered before the formation of NASH pathology showed it to have a preventive effect. Specifically, HSA-Trx was found to prevent the pathological progression to NASH by suppressing lipid accumulation, liver injury markers, and liver fibrosis. When HSA-Trx was administered during the early stage of NASH there was a marked reduction in lipid accumulation, inflammation, and fibrosis in the liver, indicating that HSA-Trx ameliorates NASH pathology. The findings indicate that HSA-Trx influences multiple pathological factors, such as oxidative stress, inflammation, and apoptosis, to elicit a therapeutic benefit. HSA-Trx also inhibited palmitic acid-induced lipotoxicity in HepG2 cells. Taken together, these results indicate that HSA-Trx has potential as a therapeutic agent for NASH pathology.

## Graphical abstract

**Figure:**
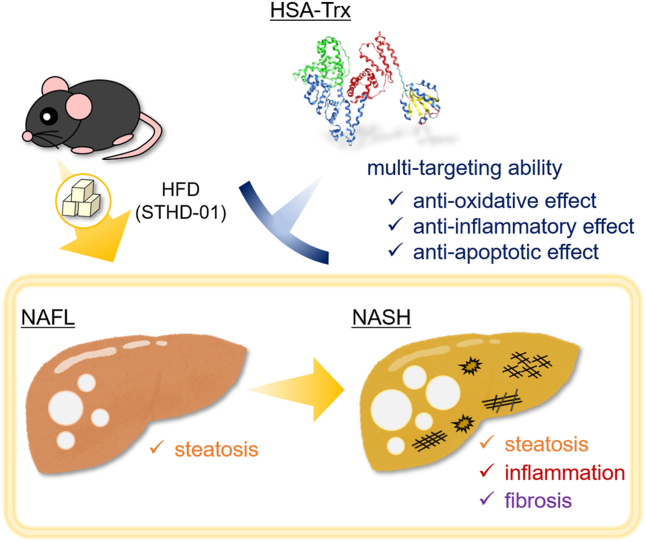

## 1 Introduction

Non-alcoholic steatohepatitis (NASH) is a progressive disease characterized by lipid accumulation, inflammation, and fibrosis in the liver, which eventually cause cirrhosis and hepatocellular carcinoma [1,2]. The prevalence of NASH is currently estimated to be about 5 % worldwide. Because NASH is associated with metabolic diseases such as obesity, the number of NASH patients is expected to further increase in the future [2]. Unfortunately, the development of therapeutic agents for NASH has proved to be problematic [3,4]. Many of these difficulties are related to the complex pathogenetic mechanisms of NASH and the irreversible progression of fibrosis formation. The “multiple parallel hits hypothesis” has been proposed to explain the pathogenesis of NASH, which is caused by the simultaneous interaction of various factors such as lipid accumulation, oxidative stress, and inflammatory response in the liver [5]. The interaction of these multiple factors leads to impaired lipid metabolism and neutrophil infiltration, which are hallmarks of NASH. These changes eventually result in liver fibrosis. Development of liver fibrosis is a predictive factor in the pathogenesis of NASH [6,7]. Thus, it is essential to suppress liver fibrosis in the treatment of NASH.

The formation of liver fibrosis in NASH is mainly triggered by the activation of hepatic stellate cells, which are also called hepatic fibroblasts, induced by persistent oxidative stress and inflammation [8]. A recent study found that apoptosis signal-regulating kinase (ASK1) activity is associated with activation of hepatic stellate cells [9]. ASK1 is a member of the MAP kinase family of enzymes and has been identified as a key factor in fibrosis formation by activating the downstream MAPK signaling pathway, leading to inflammation and apoptosis. Thus, ASK1 has been highlighted as a new therapeutic target for NASH. For example, the ASK1 inhibitor selonsertib (also known as GS4997) is in clinical development for NASH in the United States. Although the clinical trials of selonsertib alone have been terminated, clinical trials in combination with other drugs are currently ongoing [10,11]. Thus, although basic research suggests ASK1 to be a potential therapeutic target for the treatment of NASH, clinical studies indicate that ASK1 inhibition in isolation is insufficient. Simultaneous targeting of other disease progression factors could be important for the treatment of NASH. These considerations motivated us to develop a novel drug that can act comprehensively against multiple disease progression factors involved in the pathogenesis of NASH.

Here, we focused on thioredoxin (Trx), an endogenous redox regulator, as a potential therapeutic agent for NASH. Trx is important in maintaining homeostasis by regulating molecular redox reactions [12,13]. In addition to its anti-oxidative effect, it also has an anti-inflammatory effect by inhibiting neutrophil migration, infiltration and extravascular leakage at inflammatory sites [14], and by acting directly on infiltrating macrophages to induce macrophage M2 conversion [15]. Interestingly, endogenous Trx also has anti-apoptotic effects by acting as a negative regulator for ASK1 by inhibiting ASK1 activation [16]. In clinical cases, it has been reported that serum Trx levels are increased in patients with NASH by comparison to healthy controls, indicating that Trx may elicit a hepatoprotective function by increasing its expression during hepatic injury [17].

However, Trx is a low molecular weight protein of approximately 12 kDa and is rapidly excreted from the circulatory system by glomerular filtration. To resolve this issue, we produced recombinant human serum albumin-fused Trx (HSA-Trx). The HSA-Trx fusion was specifically designed to facilitate long-acting properties. Our previous study demonstrated that HSA-Trx has 10-fold longer blood retention than Trx alone [18], as well as superior therapeutic effects against various diseases [[19], [20], [21], [22]].

Here, we analyzed high-fat diet (HFD)-induced NASH model mice administered HSA-Trx to assess its preventive and therapeutic effect. To better understand the underlying molecular mechanism of HSA-Trx we also performed a series of experiments using cultured cells.

## 2 Materials & methods

### 2.1 Preparation and purification of HSA-Trx

The production and purification of HSA-Trx was performed according to previously report [22]. The *Pichia* Expression Kit was purchased from Invitrogen (Carlsbad, CA, USA).

### 2.2 Mice model of HFD-induced NASH

Male C57BL/6J mice (SLC, Shizuoka, Japan) of 9-weeks of age were randomized according to body weight. The NASH model mice were induced by feeding STHD-01 as HFD (EA Pharma, Tokyo, Japan) for 4–6 weeks [23]. At 2 or 4 weeks after starting the HFD, HSA-Trx (200 nmol/kg) was intravenously administered twice per week for 2 weeks (HFD + HSA-Trx group). The control group was injected with an equivalent amount of PBS (10 ml/kg) (HFD + PBS group).

### 2.3 Measurement of plasma ALT and AST levels

Blood was collected from mice at 2, 4 and 6 weeks after HFD feeding, and then centrifuged to obtain the plasma. Samples of plasma were assayed for alanine aminotransferase (ALT) and aspartate aminotransferase (AST) activity as markers of hepatic damage.

### 2.4 Analysis of hepatic triglyceride, cholesterol, and fatty acid composition

After collecting the liver from the mouse, lipid extraction was performed following a previously reported methodology [24]. This sample was assayed for triglyceride and cholesterol using a Triglyceride E Test and Cholesterol E Test (FUJIFILM Wako Pure Chemical Industries, Ltd.), respectively. Fatty acid composition was measured by GC–MS [25].

### 2.5 Histological analysis

H&E staining and Sirius red staining were performed as described in our previous study [24]. Liver sections were prepared with a thickness of 4 μm and observed using a BZ-X710 microscope Images with 10–14 fields collected for each mouse were randomly acquired and quantified.

### 2.6 Immunohistochemical analysis

Nitrotyrosine, an oxidative stress marker, and myeloperoxidase (MPO), a neutrophil marker, were evaluated by immunohistochemical staining according to the procedure described in our previous report [21]. Images with 10–14 fields collected for each mouse were randomly acquired and quantified. Details of the antibodies used in this analysis are shown in Supplemental Table 1.

### 2.7 Immunofluorescence staining

TUNEL staining was performed to count apoptosis-positive cells for fluorescence as described in our previous study [26]. Details of the antibodies used in this analysis are shown in Supplemental Table 1.

### 2.8 qRT-PCR

Isolation of total RNA from the liver and qRT-PCR were performed as previously described [22]. Sequences of the oligonucleotide primers are given in Supplemental Table 2.

### 2.9 Immunoblotting

We performed immunoblotting using the method described in a previous report [22]. Details of the antibodies used in this analysis are shown in Supplemental Table 1. Each band was detected by LAS 4000 mini (GE Healthcare Ltd, Amersham, UK) and quantified with ImageJ software.

### 2.10 Cell culture and treatment

HepG2 cells were cultured in RPMI medium supplemented with 10 % fetal bovine serum and 1 % penicillin-streptomycin in a 5 % CO_2_/water-saturated incubator at 37 °C. Palmitic acid (PA) (Sigma-Aldrich, St Louis, MO, USA) was mixed with defatted albumin solution in PBS (pH 7.4) at a 7:1 M ratio at 37 °C. The mixture was passed through a 0.2 μm filtration unit and then added to HepG2 cells at 200 μM to induce the *in vitro* model of lipotoxicity.

### 2.11 Measurement of intracellular ROS production

HepG2 cells were seeded in a 96-well plate at 1.0 × 10^4^ cells/well and incubated for 24 h. HSA-Trx and HSA were added 60 min before PA treatment. Intracellular ROS was measured 6 h after PA treatment using the CM-H_2_DCFDA (Invitrogen, Waltham, MA, USA).

### 2.12 In vitro qRT-PCR

HepG2 cells were seeded in a 12-well plate at 1.0 × 10^5^ cells/well and incubated for 24 h. HSA-Trx (0.5 μM) was added 60 min before PA stimulation. Isolation of total RNA from the cell lysate and the qRT-PCR were performed as previously described [27]. Sequences of the oligonucleotide primers are given in Supplemental Table 2.

### 2.13 Statistical analyses

All experimental data are shown as the mean ± standard error. Statistical analysis software used GraphPad Prism 9 (GraphPad Software, CA, USA). The means for more than two groups were compared by one-way ANOVA, and multiple comparison tests were performed by the Turkey method. A value of P < 0.05 was considered to be significant.

## 3 Results

### 3.1 The preventive effect of HSA-Trx on the pathological progression to NASH

**Fig. 1:**
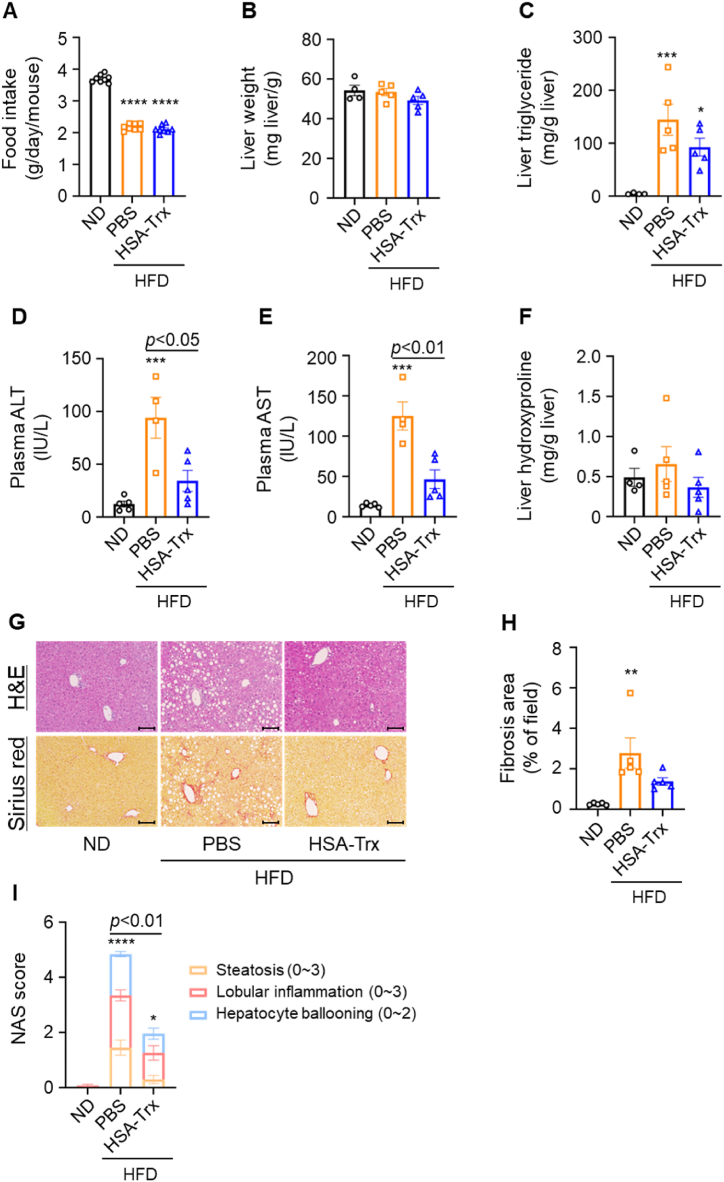
The preventive effect of HSA-Trx on the pathological progression to NASH (A) Food intake was measured twice a week for 4 weeks after feeding HFD. (B) Liver weight was calibrated for body weight at 4 weeks after feeding HFD. (C) Hepatic triglyceride was calibrated for liver weight. (D) Plasma ALT and (E) Plasma AST levels were measured 2 weeks after the administration of HSA-Trx. (F) Hepatic hydroxyproline was calibrated for liver weight. (G) Histological analysis; H&E staining (upper panel) and Sirius red staining (lower panel). Original magnification: × 200. Scale bars represent 100 μm. (H) NAS score was quantified with HE staining. (I) Quantification of fibrosis area determined after Sirius red staining. Results are the means ± S.E. (n = 4–5). *p < 0.05, **p < 0.01, ***p < 0.001, ****p < 0.0001 compared with the ND group. (For interpretation of the references to colour in this figure legend, the reader is referred to the Web version of this article.)

Based on a previous report [23], we used the NASH model mice induced by feeding STHD-01 as a HFD and confirmed that NAFL pathology was observed with lipid accumulation after 2 weeks followed by NASH pathology with liver injury and fibrosis after 4 weeks (Fig. S1). To examine the preventive effect of HSA-Trx on the pathological progression to NASH, HSA-Trx was administered intravenously at 200 nmol/kg twice a week for 2 weeks (HFD + HSA-Trx group) while the mice were being fed a HFD (Fig. S2A). The control group was administered PBS (HFD + PBS group), and the healthy group was fed a normal diet (ND) (ND group). Food intake decreased in the HFD + PBS group as compared to the ND group, but there was no significant difference between the PBS and HSA-Trx groups (Fig. 1A). Liver and adipose tissue weight did not change between the ND and HFD groups, nor were these metrics affected by HSA-Trx administration (Fig. 1B, S2C–F). However, for the HFD + PBS group there was an increase in the liver triglyceride (TG) level, which represents lipid accumulation, but this increase was significantly suppressed by HSA-Trx administration (Fig. 1C). Furthermore, the plasma ALT and AST levels as markers of liver injury were significantly increased in the HFD + PBS group, while HSA-Trx administration significantly suppressed these changes (Fig. 1D and E). Liver hydroxyproline as a quantification of liver fibrosis was also significantly suppressed in the HSA-Trx group (Fig. 1F). Histological analysis, conducted after HE staining, showed a significant decrease in the accumulation of lipid droplets, ballooning of hepatocytes, and infiltration of inflammatory cells in the HSA-Trx group (Fig. 1G upper panel). The HSA-Trx group also displayed significantly reduced fibrotic areas as observed after Sirius red staining (Fig. 1G lower panel) and its subsequent quantification (Fig. 1H). The NAS score, which evaluates steatosis, lobular inflammation, and hepatocyte ballooning [28], showed the PBS group to be in the early stages of NASH (Fig. 1I). By contrast, the HSA-Trx group was not considered to be in NASH and was judged to be in NAFL (Fig. 1I). These results indicated that HSA-Trx prevented HFD-induced NASH progression.

### 3.2 The therapeutic effect of HSA-Trx on NASH pathology

**Fig. 2:**
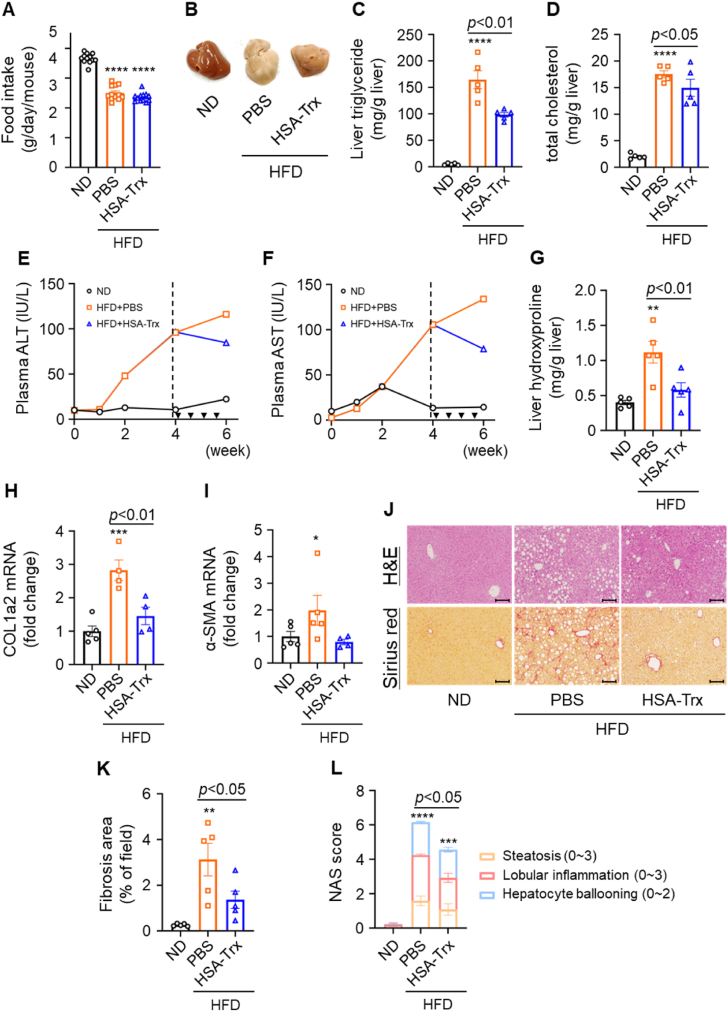
The therapeutic effect of HSA-Trx on NASH pathology (A) Food intake was measured twice a week for 6 weeks after feeding HFD. (B) Macroscopic examination of livers from each group. (C) Hepatic triglyceride and (D) total cholesterol were calibrated for liver weight at 6 weeks after feeding HFD. (E) Plasma ALT and (F) plasma AST levels were measured every 2 weeks for 6 weeks. (G) Hepatic hydroxyproline was calibrated for liver weight. (H) Collagen 1a2 and (I) α-SMA mRNA expression levels as determined by qRT-PCR. Results were corrected for each ND group and the fold change calculated. (J) Histological analysis; H&E staining (upper panel) and Sirius red staining (lower panel). Original magnification: × 200. Scale bars represent 100 μm. (K) NAS score was quantified with HE staining. (L) Quantification of fibrosis area determined after Sirius red staining. Results are the means ± S.E. (n = 5). *p < 0.05, **p < 0.01, ***p < 0.001, ****p < 0.0001 compared with the ND group. (For interpretation of the references to colour in this figure legend, the reader is referred to the Web version of this article.)

NASH pathology as evidenced by liver injury and fibrosis formation after 4 weeks of HFD feeding was confirmed as shown in Fig. S1. Next, the therapeutic effect of HSA-Trx on NASH in this model mouse was examined. HSA-Trx was administered intravenously at 200 nmol/kg twice a week from week 4 to week 6 of HFD feeding (Fig. S3A). Food intake, liver weight and fat weight did not change between the HFD + PBS and HFD + HSA-Trx groups (Fig. 2A, S3C–F). Macroscopic examination of the liver revealed whitening in the HFD + PBS group, which partially recovered following HSA-Trx administration (Fig. 2B). Liver TG and liver cholesterol (Chol) levels were significantly decreased in the HSA-Trx group as compared to the PBS group (Fig. 2C and D). Interestingly, administration of HSA-Trx reduced plasma ALT and AST levels even after the onset of NASH pathogenesis, suggesting HSA-Trx could restore liver injury (Fig. 2E and F). We also evaluated liver fibrosis as an important predictive factor of NASH status. Remarkably, hydroxyproline levels in the liver and fibrosis markers, such as collagen expression and α-SMA expression, were significantly improved in the HSA-Trx group (Fig. 2G–I). These observations suggest that HSA-Trx can improve liver fibrosis in NASH pathology. Furthermore, histological evaluation by HE staining and Sirius red staining showed significantly reduced lipid droplet accumulation and fibrotic areas after HSA-Trx administration (Fig. 2J and K). These findings were further supported by quantification using the NAS score (Fig. 2L). Thus, HSA-Trx appears to elicit a therapeutic effect on HFD-induced NASH pathology.

### 3.3 Mechanism of the therapeutic effect of HSA-Trx on NASH pathology

**Fig. 3:**
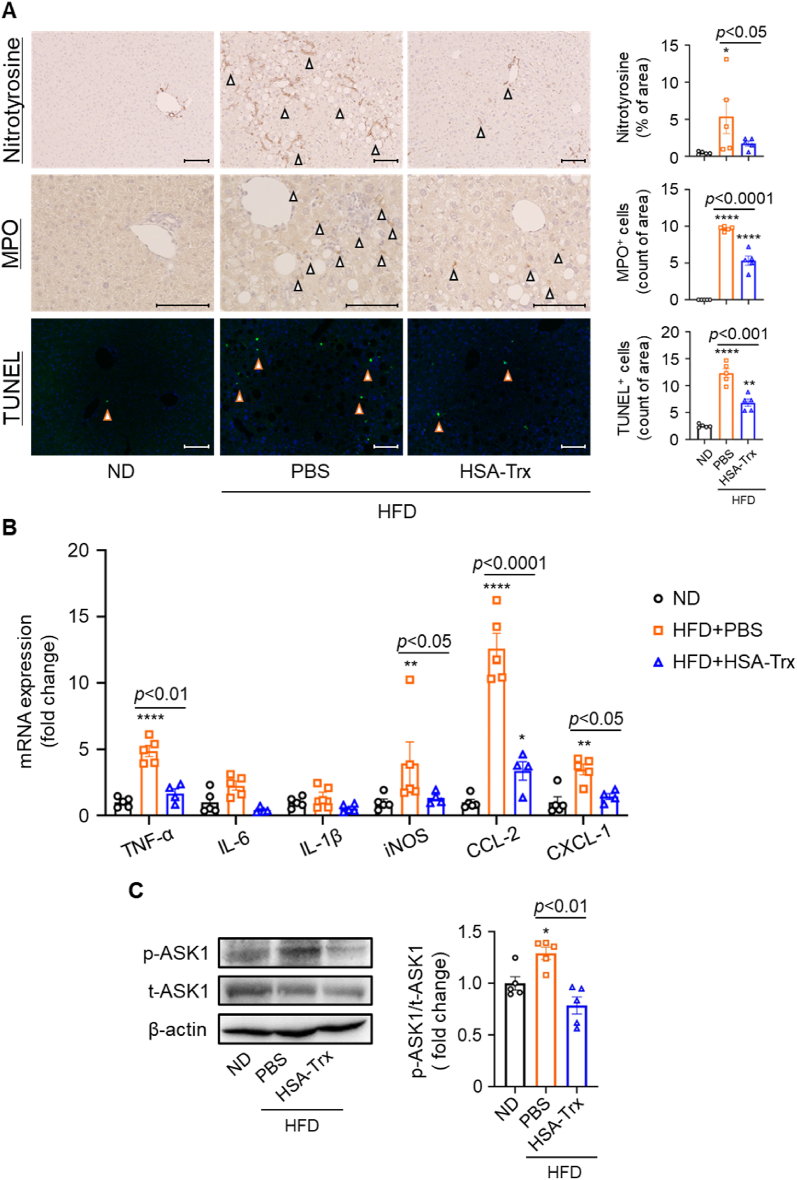
Mechanism of therapeutic effect of HSA-Trx in NASH pathology (A) Immunohistochemical assays for nitrotyrosine (upper panel) and MPO (middle panel). Immunofluorescence assay using TUNEL (lower panel) as quantified with a BZ analyzer. Triangles indicate the respective target marker in the corresponding image field. Original magnification: × 200 or × 400. Scale bars represent 100 μm. (B) Relative mRNA expression level of inflammatory-related genes (TNF-α, IL-6, IL-1β, iNOS, CCL-2, CXCL-1). Results were corrected for each ND group and the fold change calculated. (C) Immunoblot analysis was performed. Bands corresponding to phosphorylated ASK1 (p-ASK1), total ASK1 (t-ASK1), and β-actin are shown (left-hand side). Quantification of each band was performed using Image J software (right-hand side). Results are the means ± S.E. (n = 5). *p < 0.05, **p < 0.01, ****p < 0.0001 compared with the ND group.

Next, we investigated the potential mechanisms by which HSA-Trx suppresses the progression of NASH pathology, particularly its effect on oxidative stress, inflammation, and apoptosis. First, immunohistochemical analysis was used to evaluate the level of nitrotyrosine, which accumulates in the liver as a result of oxidative stress [29]. The HFD + PBS group showed a significant accumulation of nitrotyrosine in the liver, but this was markedly suppressed in the HFD + HSA-Trx group (Fig. 3A upper panel). Increased mRNA expression of inflammatory cytokines and chemokines involved in the inflammatory response, including TNF-α, IL-6, CCL-2 and CXCL-1 as observed in the HFD + PBS group, was significantly suppressed in the HFD + HSA-Trx group (Fig. 3B). The neutrophilic infiltrate is a characteristic feature of NASH and is involved in inflammation exacerbation [8,30], which was evaluated by MPO immunostaining. The increased number of MPO-positive cells observed in the HFD + PBS group was significantly suppressed in the HFD + HSA-Trx group (Fig. 3A middle panel). This finding suggests that HSA-Trx treatment suppressed a proinflammatory event, which contributed to the amelioration of HFD-induced NASH pathogenesis. We also evaluated the activation of ASK1, which is important for apoptosis signaling. In the HFD + PBS group, the expression of phosphorylated ASK1 was significantly increased as compared to the ND group (Fig. 3C). In the HFD + HSA-Trx group, ASK1 was phosphorylated to the same level as the ND group (Fig. 3C). Consistent with this result, the number of apoptosis-positive cells, as revealed by TUNEL staining, was significantly increased in the HFD + PBS group and significantly decreased in the HFD + HSA-Trx group (Fig. 3A lower panel).

### 3.4 Effect of HSA-Trx on hepatic lipid metabolism

**Fig. 4:**
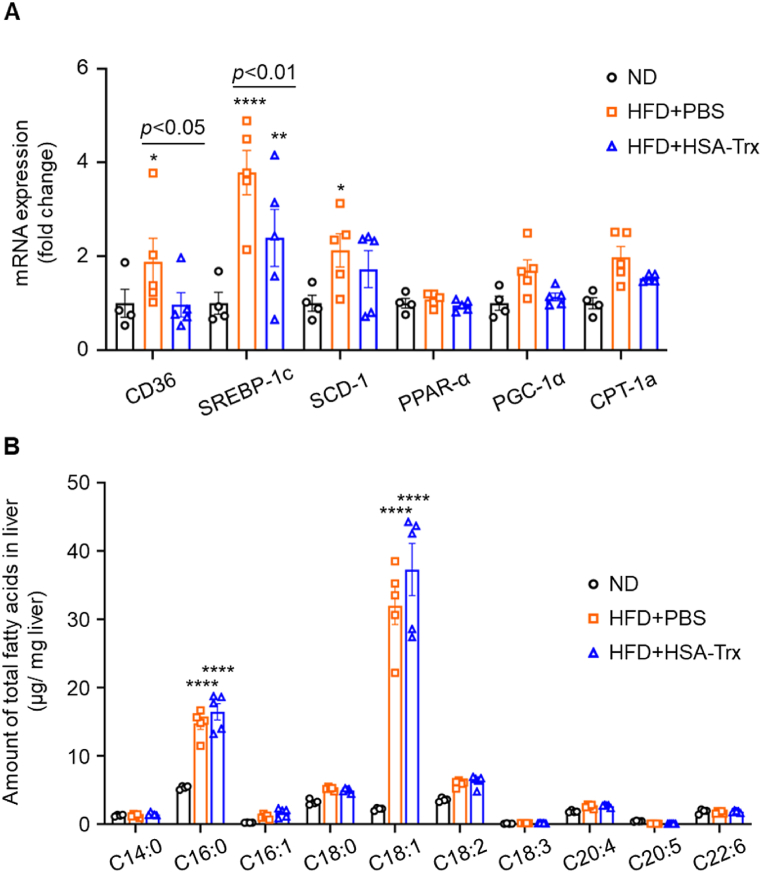
Effect of HSA-Trx on hepatic lipid metabolism: (A) Relative mRNA expression of fatty acid metabolism-related genes (CD36, SREBP-1c, SCD-1, PPAR-α, PGC-1α, CPT-1a). Results were corrected for each ND group and the fold change calculated. (B) Hepatic fatty acid composition was determined using GC-MS at 4 weeks after HFD feeding. Results are the means ± S.E. (n = 4–5). *p < 0.05, **p < 0.01, ****p < 0.0001 compared with the ND group.

As shown in Fig. 1C–. 2C and D, the HFD + HSA-Trx group showed improved hepatic lipid accumulation, indicating that HSA-Trx affects lipid metabolism. Hence, we evaluated the expression of factors involved in fatty acid uptake, synthesis, and degradation. The expression of CD36, which is involved in fatty acid uptake [31], together with SREBP-1c and SCD-1 as related factors of fatty acid synthesis [32,33], were each significantly increased in the HFD + PBS group but suppressed in the HFD + HSA-Trx group (Fig. 4A). The expression levels of PGC-1α and CPT-1a, which are associated with fatty acid degradation (β-oxidation) [34,35], were also significantly increased in the HFD + PBS group and suppressed in the HFD + HSA-Trx group (Fig. S4A). Furthermore, the fatty acid composition of the liver did not change between the HFD + PBS and HFD + HSA-Trx groups (Fig. 4B). Taken together, these data suggest that HSA-Trx can ameliorate fatty acid-induced lipotoxicity without affecting the composition of the fatty acid.

### 3.5 Inhibitory effect of HSA-Trx on palmitate-induced lipotoxicity in HepG2 cells

**Fig. 5:**
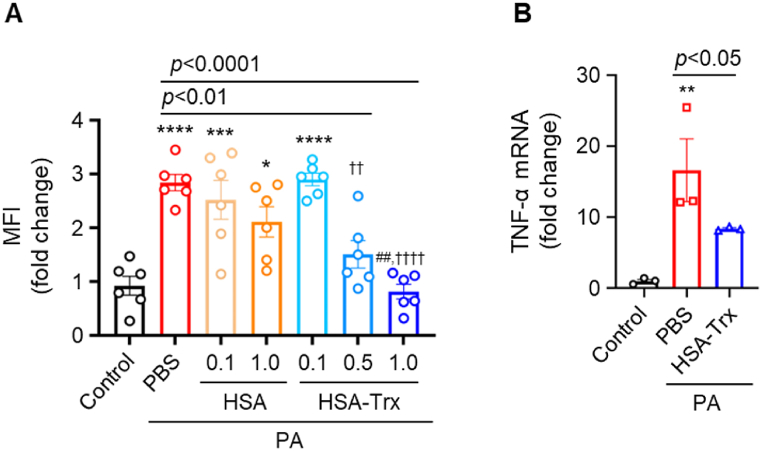
Inhibitory effect of HSA-Trx on palmitate-induced lipotoxicity in HepG2 cells (A) Intracellular ROS production was measured by Mean Fluorescence Intensity (MFI) with CM-H_2_DCFDA. Results are the means ± S.E. (n = 6). *p < 0.05, ***p < 0.001, ****p < 0.0001 compared with control. ^##^*p* < 0.01 compared with PA-HSA (1.0 μM). ^††^*p* < 0.01, ^††††^*p* < 0.0001 compared with PA-HSA-Trx (0.1 μM). (B) TNF-α mRNA expression levels as determined by qRT-PCR. Results were corrected for the control group and the fold change calculated. Results are the means ± S.E. (n = 3). **p < 0.01 compared with the control.

To further investigate the inhibitory mechanism of HSA-Trx on NASH progression, we evaluated the effect of HSA-Trx on fatty acid-induced oxidative stress and inflammation in HepG2 cells. As previously reported [36], we mimicked hepatocyte injury by lipotoxicity using HepG2 cells treated with palmitic acid (PA) (C16:0). Reactive oxygen species (ROS) production was significantly increased for both PA + PBS and PA + HSA groups by comparison to the control (no PA). However, HSA-Trx administration significantly suppressed ROS production in a concentration-dependent manner (Fig. 5A). Moreover, 1.0 μM HSA-Trx suppressed ROS production to the same level as that of the control. With regard to the inflammatory response, the increased expression of TNF-α in the PA + PBS groups were significantly suppressed by comparison to the HSA-Trx treated cells (Fig. 5B).

## 4 Discussion

Currently, NASH is considered to be a hepatic lesion of metabolic syndrome, and the number of patients is increasing along with the increase in the number of obese patients [1]. Here, we focused on Trx, which has multiple pharmacological properties such as anti-oxidative, anti-inflammatory, and anti-apoptotic effects [12,15,16]. Specifically, we produced HSA-Trx fusion protein with the aim of overcoming the low blood retention of Trx. Here, we evaluated the therapeutic effect of HSA-Trx against HFD-induced NASH pathology. The results showed that HSA-Trx not only had a preventive effect on HFD-induced NASH but also a therapeutic effect on the progression of NASH from an early stage. STHD-01 used in this study is a high-fat diet designed to explore the effectiveness of drugs on NASH [23]. In particular, STHD-01 can establish the characteristics of NASH, such as hepatic lipid accumulation, inflammation, and liver fibrosis, from an earlier period by comparison to other high-fat diets. Indeed, the mice fed STHD-01 showed evidence of lipid accumulation in the liver after only 2 weeks, indicating the establishment of NAFL pathology. At 4 weeks after HFD feeding, the mice showed a further increase of lipid accumulation in the liver, elevated ALT levels, and fibrosis, indicating the onset of NASH pathology. Moreover, at 6 weeks after HFD feeding, the mice showed a further exacerbation of lipid accumulation and increased levels of ALT/AST along with medium levels of liver fibrosis, indicating that the NASH pathology was more progressive. Although food intake was reduced as compared to the ND group, the approximate daily caloric intake was almost equal to that of the ND group (Fig. S1).

**Fig. 6:**
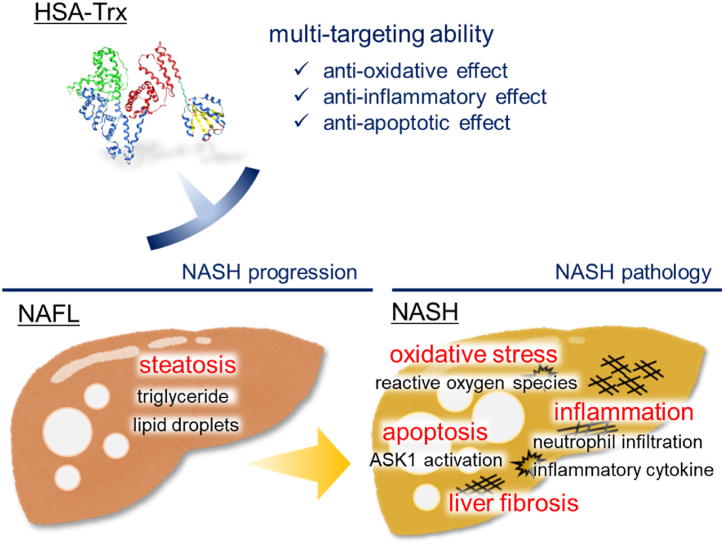
The multiple targets of HSA-Trx on the HFD-induced NASH model.

Initially, we examined the preventive effect of HSA-Trx on the transition from NAFL to NASH pathology. Our findings showed that HSA-Trx treatment significantly restricted an increase in both lipid accumulation and liver injury markers, as well as the formation of liver fibrosis. These findings demonstrated that HSA-Trx could prevent the progression of NAFL to NASH pathology (Fig. 1). Next, the therapeutic effect of HSA-Trx on NASH was also evaluated by administering HSA-Trx from 4 weeks after HFD feeding, corresponding to the initial phase of NASH pathology in the control group (HFD + PBS). Histological findings of the liver and quantification of hepatic TG and Chol levels showed that the increased lipid accumulation was significantly suppressed by HSA-Trx administration. In addition, we also found that HSA-Trx administration ameliorated the further increase in liver injury markers and exacerbation of liver fibrosis associated with the progression of NASH (Fig. 2). These results indicated that administration of HSA-Trx could ameliorate NASH progression (Fig. 6). Because this study was conducted under conditions of continuous HFD feeding, the potential effects of HSA-Trx on NASH pathology are expected to have a high impact. Unlike many other drugs that are currently being investigated for clinical application, HSA-Trx could have multiple pharmacological targets related to NASH pathology. Therefore, HSA-Trx could be an effective therapeutic agent with a wide range of applications, such as the prevention of NAFL to NASH progression or amelioration of NASH pathology.

Recently, Trx has been attracting attention as a potential therapeutic agent for several diseases, not only as a redox regulator but also as an inflammatory and apoptotic regulator [12,13,16]. The therapeutic effect of HSA-Trx on NASH pathology observed in this study are presumably due to the multiple pharmacological effects of Trx. The active site of Trx includes two cysteine residues, at positions 32 and 35, that are essential for its direct radical scavenging ability and interaction with other proteins [14,16,37,38]. Trx also exerts an indirect anti-oxidative effect through a cooperative reaction with peroxiredoxin (Prx), which is involved in the degradation of H_2_O_2_ [38,39]. In the present study, HSA-Trx treatment significantly reduced the accumulation of nitrotyrosine, which is an oxidative stress marker for NASH pathology (Fig. 3A). Moreover, HSA-Trx treatment reduced ROS production in PA-induced HepG2 cells (Fig. 5A).

Trx can also interact with macrophage migration inhibition factor (MIF), thioredoxin interacting protein (TXNIP) and ASK1 [14,16,40]. MIF is mainly produced by stress-stimulated cells and macrophages to promote the infiltration of inflammatory cells, such as neutrophils, and induce the production of proinflammatory cytokines in macrophages [41]. TXNIP is a component of the NLRP3 inflammasome complex and plays an important role in activating inflammatory pathways such as IL-1β production [40]. These factors are known to be important for the inflammatory response in the pathogenesis of NASH. It has been reported that Trx acts in an anti-inflammatory manner by interacting with these factors to suppress the inflammatory response [14]. Indeed, the anti-inflammatory effects of Trx on neutrophil infiltration and inflammatory cytokine production were confirmed in this study (Fig. 3A and B). As such, we propose that HSA-Trx suppresses not only oxidative stress but also the inflammatory response. Although the anti-inflammatory effects of Trx have also been reported to promote M2 polarization of macrophages [15], HSA-Trx had no significant effect on the expression of anti-inflammatory cytokines and M2 macrophage markers (CD163 and CD206) in this study (Fig. S4A). It is likely that the involvement of anti-inflammatory cytokines and M2 macrophage markers is influenced by the timing of evaluation. Consequently, examination at an earlier time point after HSA-Trx administration may be required.

Trx functions as an endogenous inhibitor of ASK1 by direct interaction. Specifically, the anti-apoptotic effects of Trx arise from the inhibition of ASK1 dimer formation and ASK1 phosphorylation. Importantly, the redox status of Trx affects its interaction with ASK1. When intracellular Trx is required to suppress extracellular oxidative stress or inflammation its interaction with ASK1 is lost. The released Trx migrates to the extracellular compartment and the newly released intracellular ASK1 is activated [9,16,42]. Thus, exogenous supplementation of Trx could be an effective strategy to inhibit ASK1 activation. In this study it remains unclear whether HSA-Trx interacts directly with ASK1, but the phosphorylation level of ASK1 was suppressed to the same level as observed in ND group (Fig. 3C). Apoptosis-positive cells in liver tissues were also reduced in the HSA-Trx group (Fig. 3A), suggesting that HSA-Trx might exert its anti-apoptotic effect by inhibiting ASK1 activation. There have been no reports of Trx directly inhibiting lipid accumulation, while administration of HSA-Trx significantly suppressed lipid accumulation in the liver (Fig. 1C, G, 2B-D and 2J).

The expression of CD36, a scavenger receptor, which is involved in fatty acid uptake, and SREBP-1c, a transcription factor for fatty acid synthase, is known to be upregulated in response to fatty acid-induced oxidative stress in NASH pathology [31,32]. In this study, hepatic fatty acids were abundant in the NASH model (Fig. 4B). CD36, SREBP-1c, and SCD-1 expression might be upregulated via fatty acid-induced oxidative stress. Therefore, hepatic lipid accumulation was presumably promoted by this vicious cycle (Fig. 4A). By contrast, HSA-Trx treatment suppressed oxidative stress in NASH pathology. In turn, this might suppress CD36, SREBP-1c, and SCD-1 expression, thereby lowering the accumulation of hepatic lipid. These effects support the hypothesis that multiple factors, such as oxidative stress, inflammation, and lipid metabolism, interact with each other in a complex relationship that contributes to the pathogenesis of NASH.

Endogenous Trx may contribute to the inhibitory mechanisms of HSA-Trx. Thus, we evaluated Trx expression in liver. The results showed that Trx expression was increased in NASH pathology, while the HSA-Trx group showed a similar Trx expression level to that of the ND group (Fig. S4B). This increased Trx expression could compensate for the enhanced response to oxidative stress and inflammatory reactions during NASH pathology while HSA-Trx maintained intracellular Trx expression by suppressing these events.

Liver fibrosis in NASH has been shown to be an important factor in determining long-term prognosis [7]. Thus, an improvement of liver fibrosis is an essential criterion for therapeutic strategies to address NASH pathology. A clinical goal is to restore at least one stage of liver fibrosis, but to date, no therapeutic agents have been able to achieve this aim. In this study, based on quantification of fibrotic areas by Sirius red staining and the expression levels of fibrosis markers, mice showing NASH pathology fed HFD for 6 weeks were judged to be in stages 2–3 of liver fibrosis. However, an equivalent assessment of liver fibrosis in the HSA-Trx group corresponded to stage 0–1, indicating that the clinical goal for liver fibrosis could be achieved (Fig. 2G-L). This remarkable result justifies clinical development of HSA-Trx as a potential treatment. It does not seem that HSA-Trx directly degraded liver fibrosis, but instead suppressed liver fibrosis formation by inhibiting oxidative stress, inflammation, and hepatic injury. In addition, the liver might be able to maintain normal function through hepatocyte regeneration [43], in which damaged hepatocytes are regenerated as normal new cells. In particular, the regeneration of hepatocytes requires a lot of energy, and it may be sufficient for the damaged cells to regenerate themselves using TG as an energy source in a condition with excessive TG accumulation such as NASH [43,44]. Therefore, it is possible that the effect of the HSA-Trx on lipid accumulation might be related to the conversion of excess TG into energy through this mechanism. This potential mechanism will be examined in a future study.

## 5 Conclusion

Our findings demonstrate that administration of HSA-Trx elicits preventive and therapeutic effects on HFD-induced NASH pathogenesis by exerting anti-oxidative, anti-inflammatory, and anti-apoptotic effects. HSA-Trx could be a potential therapeutic agent for NASH by targeting multiple pathological factors.

## Data availability

The datasets generated and/or analyzed during the current study are available from the corresponding author on reasonable request.

## CRediT authorship contribution statement

**Ryota Murata:** Writing – original draft, Visualization, Project administration, Methodology, Investigation, Formal analysis, Data curation, Conceptualization. **Hiroshi Watanabe:** Writing – review & editing, Supervision, Resources, Project administration, Methodology, Funding acquisition, Data curation, Conceptualization. **Ryotaro Iwakiri:** Visualization, Investigation. **Mayuko Chikamatsu:** Visualization, Investigation. **Takao Satoh:** Visualization, Investigation. **Isamu Noguchi:** Investigation. **Kengo Yasuda:** Investigation. **Ayano Nishinoiri:** Investigation. **Takuma Yoshitake:** Investigation. **Hiroto Nosaki:** Investigation. **Hitoshi Maeda:** Validation, Supervision. **Toru Maruyama:** Writing – review & editing, Supervision, Project administration.

## Declaration of competing interest

The authors declare the following financial interests/personal relationships which may be considered as potential competing interests:Hiroshi Watanabe reports financial support was provided by Kaken Pharmaceutical Co Ltd. Hiroshi Watanabe reports financial support was provided by 10.13039/100007449Takeda Science Foundation. Hiroshi Watanabe reports financial support was provided by KM Biologics Co Ltd. If there are other authors, they declare that they have no known competing financial interests or personal relationships that could have appeared to influence the work reported in this paper.
