# Supplementary material for: Albumin-fused thioredoxin ameliorates high-fat diet-induced non-alcoholic steatohepatitis
Source: Heliyon. 2024 Feb 2;10(3):e25485. doi: 10.1016/j.heliyon.2024.e25485 (PMC10861950; doi:10.1016/j.heliyon.2024.e25485)
Supplement: Multimedia component 1 [file mmc1.docx]

**Albumin-fused thioredoxin ameliorates high-fat diet-induced non-alcoholic steatohepatitis**

Ryota Murata, Hiroshi Watanabe, Ryotaro Iwakiri, Mayuko Chikamatsu, Takao Satoh, Isamu Noguchi, Kengo Yasuda, Ayano Nishinoiri, Takuma Yoshitake, Hiroto Nosaki, Hitoshi Maeda, Toru Maruyama

**Supplemental Information**

**Supplemental Figure 1. Preparation of HFD-induced NASH model mice:**

(A) Experimental protocol for the preparation of HFD-induced NASH model mice: C57BL/6 J mice (8-week-old, male) were fed a normal diet (ND) or high-fat diet (HFD: STHD-01). (B) Body weight was monitored during the 6 weeks of HFD feeding. (C) Food intake was measured twice a week for 6 weeks after feeding HFD. (D) Liver triglyceride was calibrated for liver weight (E) Plasma ALT level was measured twice a week for 6 weeks after feeding HFD. (F) Histological analysis; H&E staining (upper panel) and Sirius red staining (lower panel). Original magnification: ×200. Scale bars represent 100 μm. (G) Quantification of fibrosis area was determined after Sirius red staining. Results are the means ± S.E. (n=4-5). **p*<0.05, ***p*<0.01, ****p*<0.001, *****p*<0.0001 compared with the ND group.


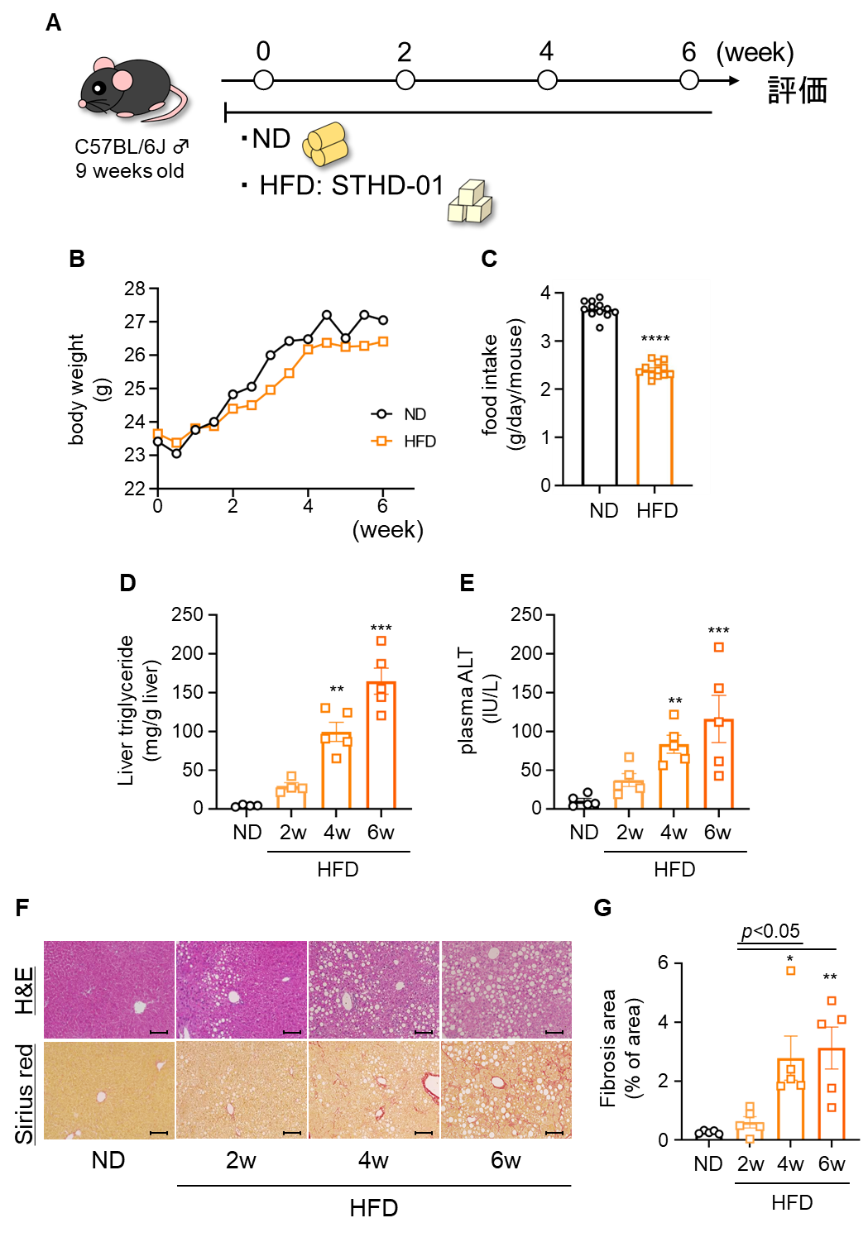


**Supplemental Figure 2. The preventive effect of HSA-Trx on the pathological progression to NASH (additional data):**

(A) Experimental protocol for evaluating the preventive effect of HSA-Trx on HFD-induced NASH model mice. Mice were randomized at 2 weeks of HFD feeding, and PBS (10 mL/kg) or HSA-Trx (200 nmol/kg) was administered intravenously twice a week for 2 weeks. (B) Body weight was monitored during the 4-week period of HFD feeding. (C) Liver weight, (D) epididymal white adipose tissue (eWAT) weight, (E) inguinal subcutaneous white adipose tissue (iWAT) weight and (F) interscapular brown adipose tissue (iBAT) weight were calibrated for body weight at 4 weeks after feeding HFD. Results are the means ± S.E. (n=4-5).


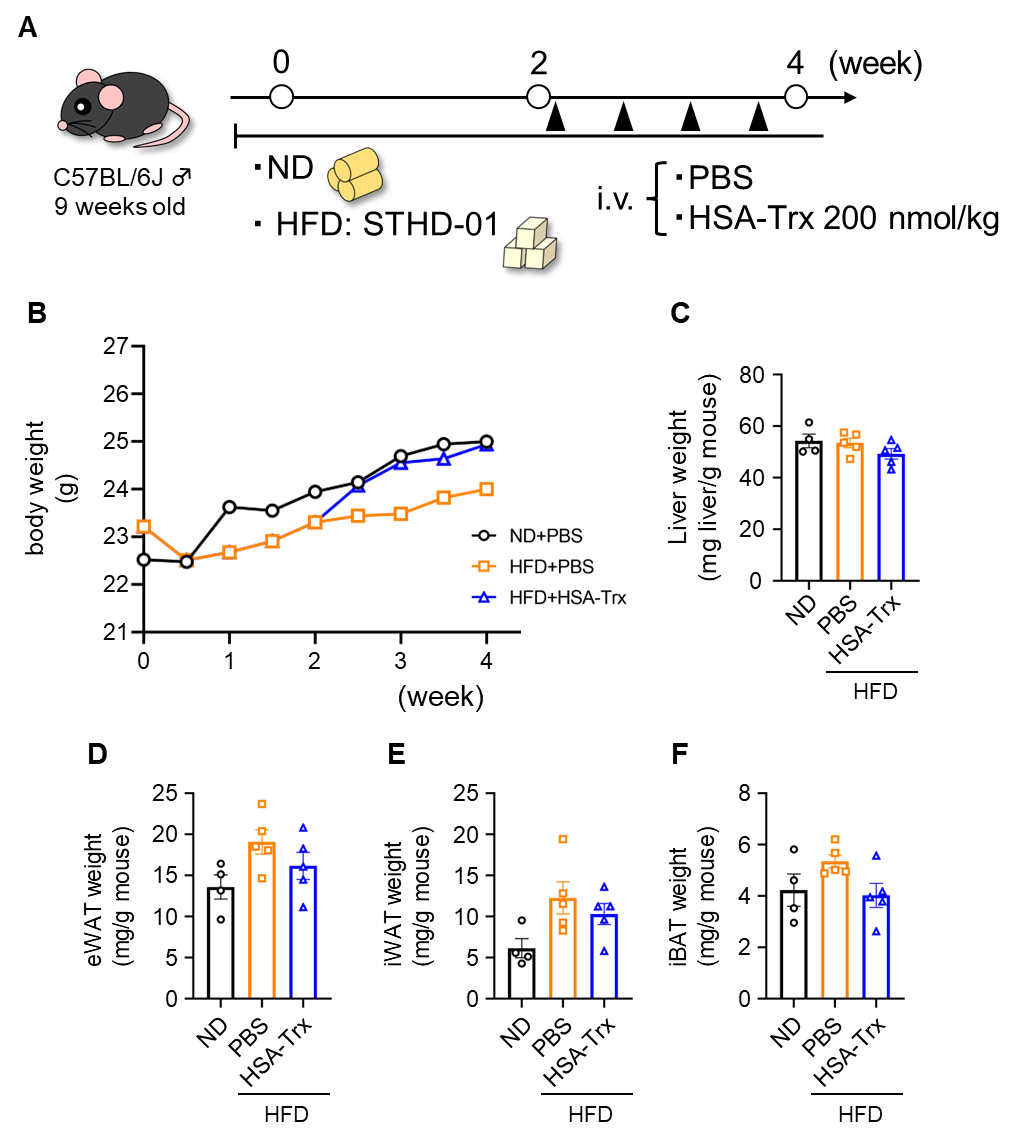


**Supplemental Figure 3. The therapeutic effect of HSA-Trx on NASH pathology (additional data):**

(A) Experimental protocol for evaluating the therapeutic effect of HSA-Trx on HFD-induced NASH model mice. Mice were randomized at 4 weeks of HFD feeding, and PBS (10 mL/kg) or HSA-Trx (200 nmol/kg) was administered intravenously twice a week for 2 weeks. (B) Body weight was monitored during the 6 weeks of HFD feeding. (C) Liver weight, (D) eWAT weight, (E) iWAT weight and (F) iBAT weight were calibrated for body weight at 6 weeks after feedingHFD. Results are the means ± S.E. (n=5).


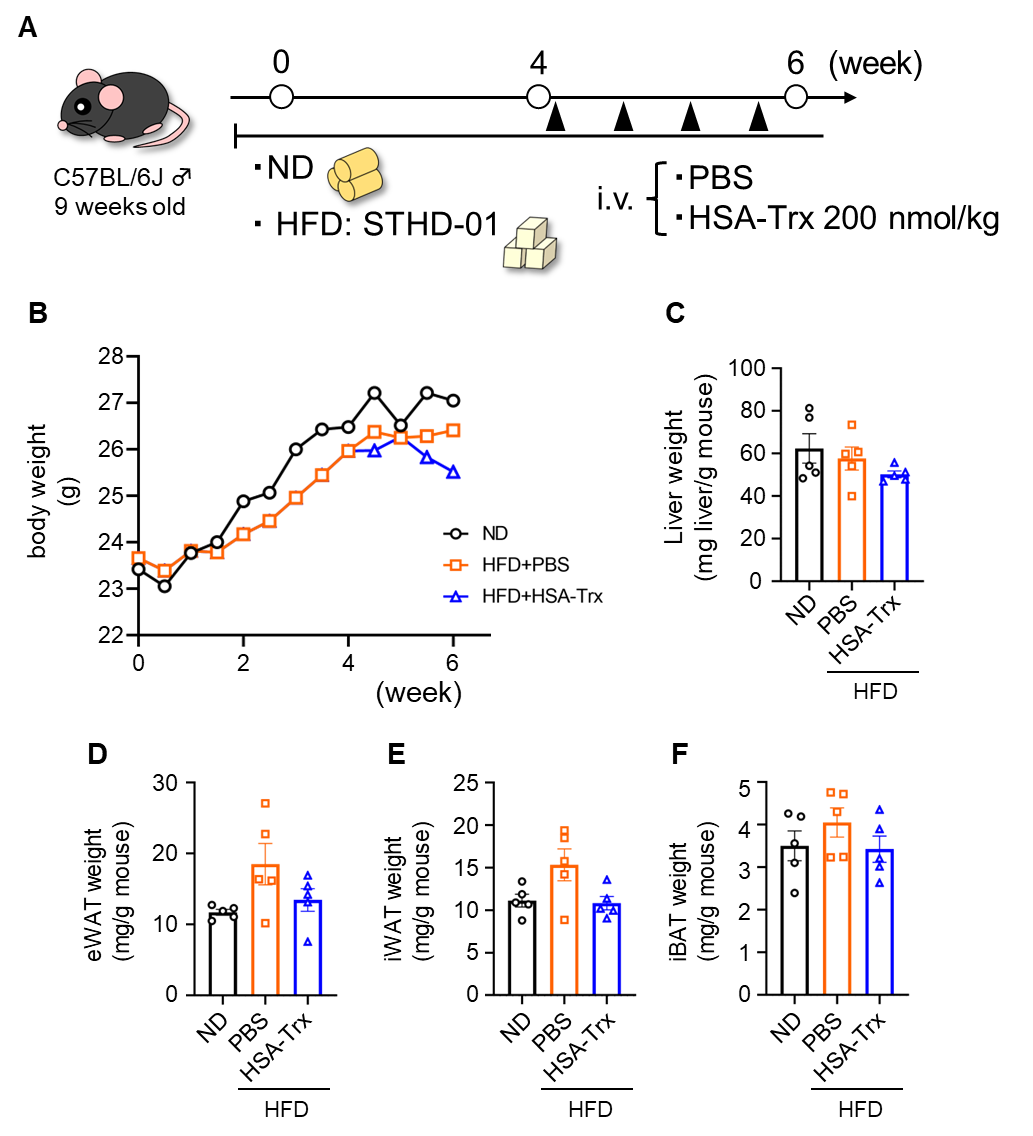


**Supplemental Figure 4. Mechanism of the therapeutic effect of HSA-Trx** **for NASH pathology (additional data):**

(A) Relative mRNA expression of anti-inflammatory-related genes (IL-4, IL-10, IL-13, CD163, CD206). Results were corrected for each ND group and the fold change calculated. (B) Immunoblot analysis was performed. Bands corresponding to Trx and β-actin are shown (left-hand side). Quantification of each band was performed using Image J software (right-hand side). Results are the means ± S.E. (n=5). **p*<0.05, ****p*<0.001, *****p*<0.0001 compared with the ND group.


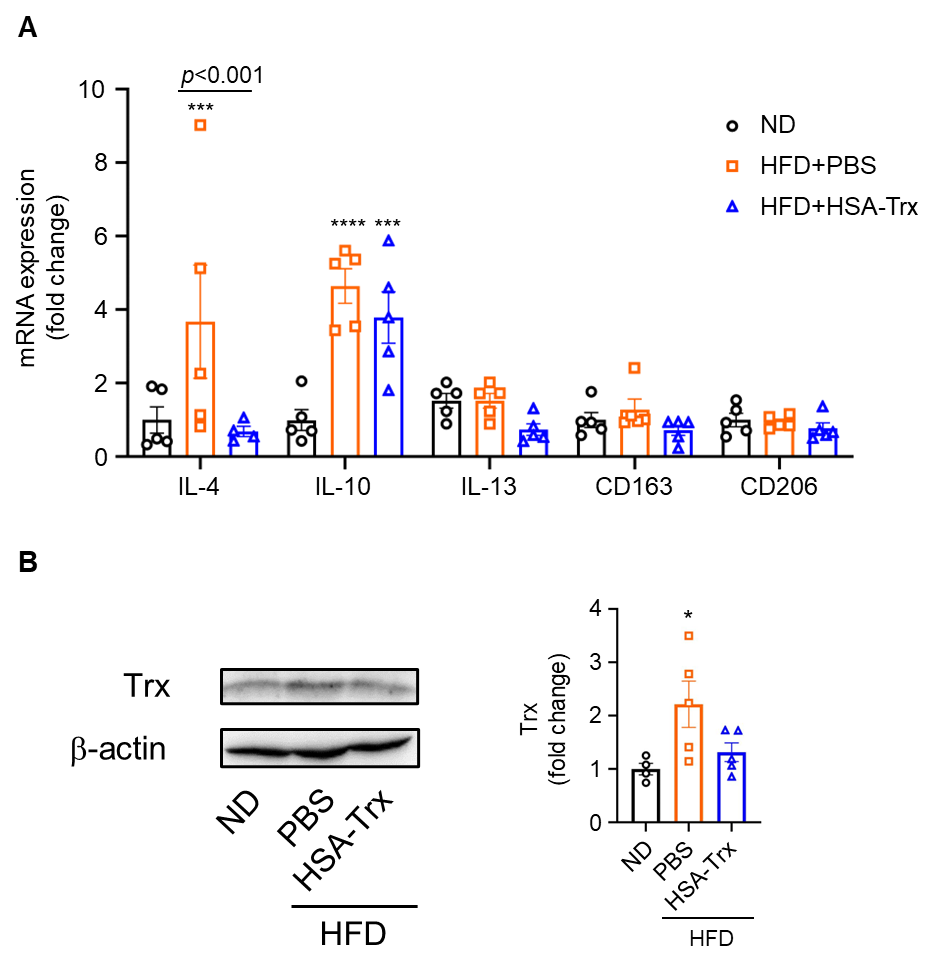


**Supplemental Figure 5. Complete Immunoblot images before cropping into the final results figures.** Cropped areas are highlighted by a red box.

**
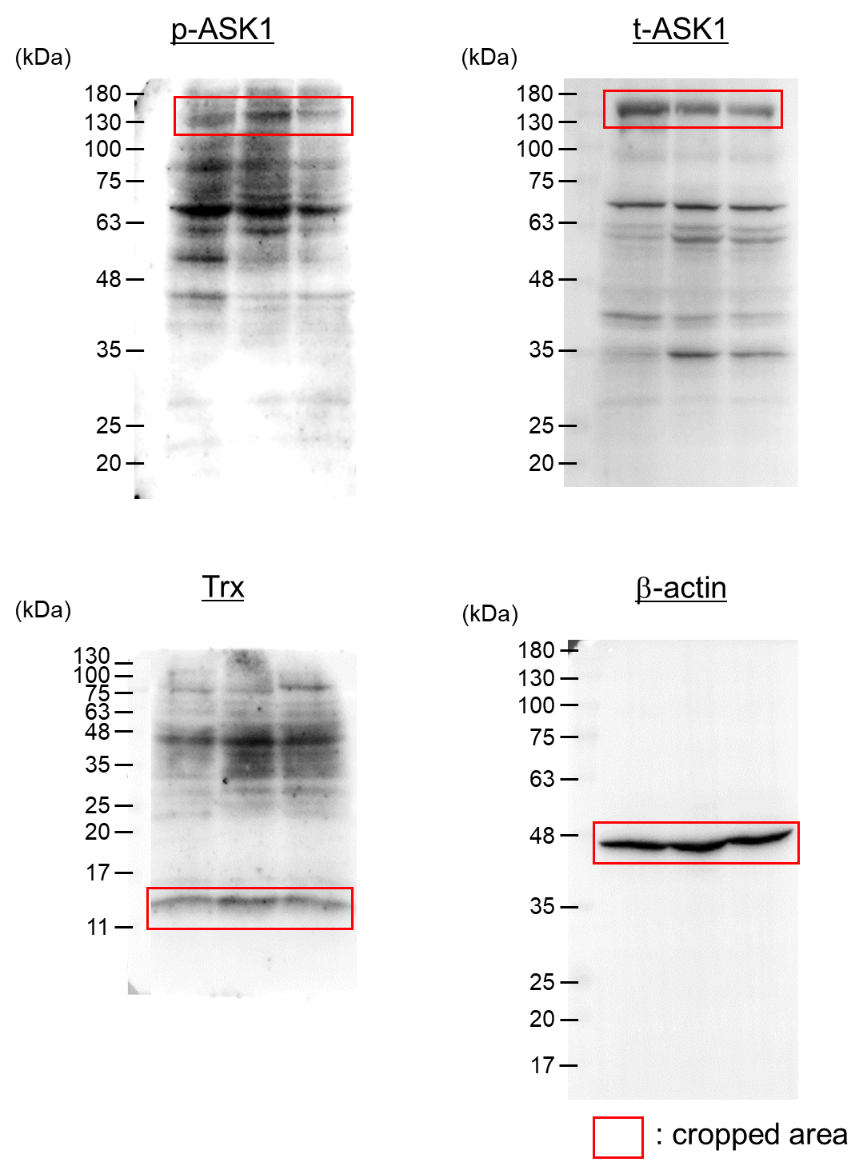
**

**Supplemental Table 1.** Sequences of oligonucleotide primer pairs used for mRNA detection.

Primers (mouse)

| **Target gene** | **Forward (5’-3’)** | **Reverse (5’-3’)** |
| --- | --- | --- |
| Collagen1a2 | GCCACCATTGATAGTCTCTCCTAAC | GCCACCATTGATAGTCTCTCCTAAC |
| α-SMA | AGCCATCTTTCATTGGGATGG | CCCCTGACAGGACGTTGTTA |
| TNF-α | CATGAGCACAGAAAGCATGATCCG | AAGCAGGAATGAGAAGAGGCTGAG |
| IL-6 | TCTCTGCAAGAGACTTCCATCC | AGACAGGTCTGTTGGGAGTG |
| IL-1β | TGAGCTGAAAGCTCTCCACC | CTGATGTACCAGTTGGGGAA |
| iNOS | GTGGTGACAAGCACATTTGG | AAGGCCAAACACAGCATACC |
| CCL-2 | TGCATCTGCCCTAAGGTCTTC | AGTGCTTGAGGTGGTTGTGG |
| CXCL-1 | ACCGAAGTCATAGCCACACTC | CTCCGTTACTTGGGGACACC |
| CD36 | TGGTCAAGCCAGCTAGAAA | CCCAGTCTCATTTAGCCAC |
| SREBP-1c | CGGCGCGGAAGCTGT | TGCAATCCATGGCTCCGT |
| SCD-1 | GCTGGGCAGGAACTAGTGAG | GGTAGGGAGGATCTGGAAGC |
| PPAR-α | TTTCGGCGAACTATTCGGCTG | GGCATTTGTTCCGGTTCTTCTT |
| PGC-1α | TCTCAGTAAGGGGCTGGTTG | AGCAGCACACTCTATGTCACTC |
| CPT-1a | AGTGGCCTCACAGACTCCA | GCCCATGTTGTACAGCTTCC |
| IL-4 | GGTCTCAACCCCCAGCTAGT | GCCGATGATCTCTCTCAAGTGAT |
| IL-10 | GGACAACATACTGCTAACCGACTC | AAAATCACTCTTCACCTGCTCCAC |
| IL-13 | TGGCTCTTGCTTGCCTTGGTGG | CCATACCATGCTGCCGTTGCA |
| CD163 | ATGGGCTAACTCCAGCGCCG | GATCCATCTGAGCAGGTCACTCCA |
| CD206 | GCCAGAGACATAACAGCA | CAGGTTTCCTTTCAGTCCT |
| GAPDH | AACTTTGGCATTGTGGAAGG | ACACATTGGGGGTAGGAACA |

Primers (human)

| **Target gene** | **Forward (5’-3’)** | **Reverse (5’-3’)** |
| --- | --- | --- |
| TNF-α | TGAAAGCATGATCCGGGACG | CAGCTTGAGGGTTTGCTACAAC |
| GAPDH | GGTGAAGGTCGGAGTCAACG | ACCATGTAGTTGAGGTCAATGAAGG |

**Supplemental Table 2.** Details of antibodies used for immunoblotting and immunohistochemical analysis.

| **Antibody** | **Dilution ratio** |
| --- | --- |
| Rabbit anti-pASK1 (Cell Signaling, 3765) | 1:2000 |
| Rabbit anti-ASK1 (GeneTex, C2C3-2) | 1:2000 |
| Mouse anti-β-actin (Sigma-Aldrich, A5441) | 1:4000 |
| Goat anti-Trx (R&D SYSTEMS, AF1970) | 1:2000 |
| Rabbit anti-Nitrotyrosine (Millipore, AB5411) | 1:200 |
| Rabbit anti-MPO (Santa Cruz, sc-16128-R) | 1:200 |
| Mouse anti-rabbit IgG-HRP (Santa Cruz, sc-2357) | 1:2000 |
| Mouse-IgGk BP-HRP (Santa Cruz, sc-516102) | 1:4000 |
| Rabbit anti-goat IgG-HRP (Santa Cruz, sc-2768) | 1:2000 |

**Expanded Material and Methods:**

***Materials***

Yeast nitrogen base with ammonium sulfate but lacking amino acids was purchased from Difco Laboratories, Inc. (Detroit, MI, USA). Hipolypepton was purchased from Nippon Shinyaku (Kyoto, Japan). Aquacide II was purchased from Merck Millipore (Burlington, MA, USA). Blue Sepharose 6 Fast Flow column and HiTrap Phenyl HP column were purchased from GE Healthcare Japan (Tokyo, Japan). Sal I and RNAiso Plus were purchased from Takara Bio Inc. (Shiga, Japan). A 10% formalin neutral buffer solution was purchased from FUJIFILM Wako Pure Chemical Industries, Ltd. (Osaka, Japan). Heparin (sodium salt) was purchased from Mochida Pharmaceutical (Tokyo, Japan). 4’,6-diamidino-2-phenylindole solution was purchased from Invitrogen (Waltham, MA). Marinol was purchased from Muto Pure Chemical (Fukuoka, Japan). All reagents and solvents were of the highest commercially available specifications and used without further purification. Ion-exchanged water or Milli-Q water was used to make up aqueous solutions.

***Animal experiment***

All animal experiments involved procedures that had been previously approved by the experimental animal ethics committee at Kumamoto University and all methods were performed in accordance with the relevant guidelines and regulations.
